# 70759 Jaw-specific control of Msx1-dependent odontogenesis by Dkk2 and Sostdc1

**DOI:** 10.1017/cts.2021.447

**Published:** 2021-03-30

**Authors:** Hyuk-Jae Edward Kwon

**Affiliations:** University at Buffalo

## Abstract

ABSTRACT IMPACT: Our proposed jaw-specific control mechanism of tooth development is expected to address the site-specific prevalence of tooth agenesis in humans. OBJECTIVES/GOALS: To determine the molecular mechanisms that control jaw-specific tooth development. To identify the molecular basis of the site-specific prevalence of humans tooth agenesis cases. METHODS/STUDY POPULATION: We used three different genetically engineered mouse lines: Msx1 ^’/ ^’, Dkk2 ^’/ ^’, and Sostdc1 ^’/ ^’ mice. We used developmental mouse genetics approaches, basically generating different combinations of compound mutant mice. We examined their tooth development by using gross, histology, and mRNA expression analyses. RESULTS/ANTICIPATED RESULTS: We identified that Sostdc1, a secreted Wnt inhibitor, also plays an important role in regulating the Msx1-dependent odontogenic pathway. Sostdc1 mRNA showed similar expression patterns in the developing tooth germs between control and Msx1-null molar buds. Remarkably, by deleting the Sostdc1 gene, as well as the Dkk2 gene, in the Msx1-null background mouse, molar tooth development was rescued in the maxillary jaw, but not in the mandibular jaw. Furthermore, tooth developmental rescue could be achieved in both the maxillary and mandibular molars by combinedly deleting Dkk2 and Sostdc1 in Msx1-null mice. DISCUSSION/SIGNIFICANCE OF FINDINGS: Our study demonstrates that secreted Wnt inhibitors Dkk2 and Sostdc1 synergistically regulate the Msx1-dependent odontogenic pathway and further control early tooth morphogenesis. These mouse model will be used to further address the site-specific prevalence of tooth agenesis in humans.

